# Reliably measuring learning-dependent distractor suppression with eye tracking

**DOI:** 10.3758/s13428-024-02552-8

**Published:** 2024-12-18

**Authors:** Andy J. Kim, Laurent Grégoire, Brian A. Anderson

**Affiliations:** 1 School of Gerontology, University of Southern California, 3715 McClintock Ave, Los Angeles, CA 90089 USA; 2 Department of Psychological & Brain Sciences, Texas A&M University, College Station, TX USA

**Keywords:** Reliability, Attention capture, Distractor suppression, Visual search

## Abstract

In the field of psychological science, behavioral performance in computer-based cognitive tasks often exhibits poor reliability. The absence of reliable measures of cognitive processes contributes to non-reproducibility in the field and impedes the investigation of individual differences. Specifically in visual search paradigms, response time-based measures have shown poor test–retest reliability and internal consistency across attention capture and distractor suppression, but one study has demonstrated the potential for oculomotor measures to exhibit superior reliability. Therefore, in this study, we investigated three datasets to compare the reliability of learning-dependent distractor suppression measured via distractor fixations (oculomotor capture) and latency to fixate the target (fixation times). Our findings reveal superior split-half reliability of oculomotor capture compared to that of fixation times regardless of the critical distractor comparison, with the reliability of oculomotor capture in most cases falling within the range that is acceptable for the investigation of individual differences. We additionally find that older adults have superior oculomotor reliability compared with young adults, potentially addressing a significant limitation in the aging literature of high variability in response time measures due to slower responses. Our findings highlight the utility of measuring eye movements in the pursuit of reliable indicators of distractor processing and the need to further test and develop additional measures in other sensory domains to maximize statistical power, reliability, and reproducibility.

**Supplementary Information:**

The online version contains supplementary material available at 10.3758/s13428-024-02552-8.

## Introduction

The field of psychological science has been challenged in the past decade to improve the replicability of behavioral research based on large-scale examples of non-reproducibility (Johnson et al., 2017; Open Science Collaboration, 2012, 2015). Nosek and colleagues define reproducibility, robustness, and replicability as “testing the reliability of a prior finding” and propose that maximizing the reliability of research findings will improve research credibility and knowledge translation into application (Nosek et al., 2022). The reliability of measurements is particularly important when maximizing the power of significance tests, and measures with poor reliability are not sensitive in detecting individual differences (Zimmerman et al., 1993). Researchers have commonly utilized two types of reliability measurements: test–retest reliability and internal consistency (split-half correlation). These tests have often revealed poor reliability of behavioral measures in the field of psychological science (Clark et al., 2022; Dang et al., 2020; Draheim et al., 2019; Paap & Sawi, 2016), highlighting a need for researchers in the field to identify and develop more reliable measures (in comparison to frequently used measures derived from behavioral responses) that can be consistently employed across a range of experimental paradigms.

The critical need for a reliable measure in standardized experimental designs has been made evident from the rise in research on individual differences as a means to more accurately characterize the underlying cognitive processes observed in human performance (Brysbaert, 2024). As the practical applications of research findings are being increasingly prioritized, maximizing the transfer of scientific knowledge requires research at the individual level. For this aim, Brysbaert (2024) emphasizes the importance of not only using standardized task protocols that have norms in addition to valid and reliable measurements but also of using robust evaluations of correlation coefficients with enough participants to attain stable reliability estimates (Hajcak et al., 2017; Schönbrodt & Perugini, 2013). Researchers are now tasked with considering relevant parameters in the design stage of their experiments, including sample size estimates from power analyses and calculating the number of trials required to observe a specific effect size, which can be optimized through publicly available toolboxes (Baker et al., 2021; Draheim et al., 2019). Although the field of experimental psychology has grown to better recognize the rigorous requirements for individual difference research (Bauer, 2011), many problems still remain, such as with large online data collection efforts as a means to attain larger sample sizes (greater than 400 participants) and achieving robust correlation estimates with small effects (Cooper, 2024). Furthermore, the cost of conducting these large-scale studies is often unrealistic (Könen & Karbach, 2021) and the burden of developing and validating robust experimental tasks for individual difference research is time consuming and often not a core goal for researchers (Brysbaert, 2024). However, in practice, researchers in the field should at least validate both the internal consistency and/or test–retest reliability of their acquired measures to create a foundation to ultimately enable productive individual differences research. Acceptable reliability estimates for research are somewhat arbitrary and vary across fields even within psychological sciences, although 0.7 is a frequently used benchmark (Taber, 2018). Identifying reliable measures of cognitive processes is especially important for individual differences research, as the reliability of two measures provides an upper bound on the strength of the relationship that can be detected between them. More generally, the reliability of a measure determines the confidence with which it can be used to draw conclusions about the performance of individual participants and establish meaningful norms.

In the field of experimental psychology, researchers utilizing visual search paradigms have recently highlighted the poor reliability of measures in commonly used task paradigms, specifically using behavioral response times. Ivanov et al. (2023) investigated whether difference scores in manual response times and accuracy were reliable and could be utilized as an individual-level measure. Utilizing both split-half and test–retest reliability measurements, the authors investigated whether attention capture learned distractor suppression at a high-probability location in the visual search array and corresponding suppression of targets at the high-probability location could serve as reliable measures for investigating individual differences (Ivanov et al., 2023). Over the three measures, the authors report poor to moderate split-half reliability over response times and poor reliability over the accuracy, in addition to poor test–retest reliability with respect to both response times and accuracy. Furthermore, three studies investigating selection history effects of reward learning in visual search also reported poor test–retest reliability of behavioral response times (Anderson & Kim, 2019; Freichel et al., 2023; Garre-Frutos et al., 2024). These studies collectively identified that response time exhibits poor reliability over experience-driven attention effects. However, in Anderson and Kim (2019), value-driven oculomotor capture exhibited strong test–retest reliability, suggesting that oculomotor capture may be more sensitive and reliable in contrast to oculomotor fixation times and even more so when compared with manual response times (Anderson & Kim, 2019; Weichselbaum et al., 2018).

Therefore, in the current study, we investigated whether oculomotor measures of distractor fixations provide superior reliability compared to response time-based measures (fixation time or time to make an eye movement to the target), providing a potential solution to enable the more robust assessment of individual differences in the attentional processing of distractors. We investigated oculomotor measures in three studies containing a total of eight experiments that utilized a visual search task incorporating attention capture and/or distractor suppression. The selected studies were limited to investigating the reliability of distractor suppression in the context of selection history effects, given pessimistic findings concerning manual response time measures (Ivanov et al., 2023). We aimed to examine the reliability of oculomotor measures in visual search across multiple experimental paradigms incorporating statistical learning of a high-probability distractor location, learned value associations with the distractor in a context in which these associations lead to reduced distractor interference, and proactive distractor suppression (feature-search) vs. reactive distractor disengagement (singleton-search). Thus, we look to evaluate the reliability of oculomotor measures across numerous critical distractor comparisons. In two cases, data from both older and younger adults was available, permitting an assessment of the reliability of oculomotor measures as a function of age. Based on the findings of Anderson and Kim (2019), we hypothesize that the reliability of oculomotor capture measures will be superior to that of measures involving fixation time, and that these oculomotor measures will also demonstrate high reliability that is superior to the characteristically low reliability associated with manual response time measures as observed in the literature.

## Methods

### Datasets

**Fig. 1:**
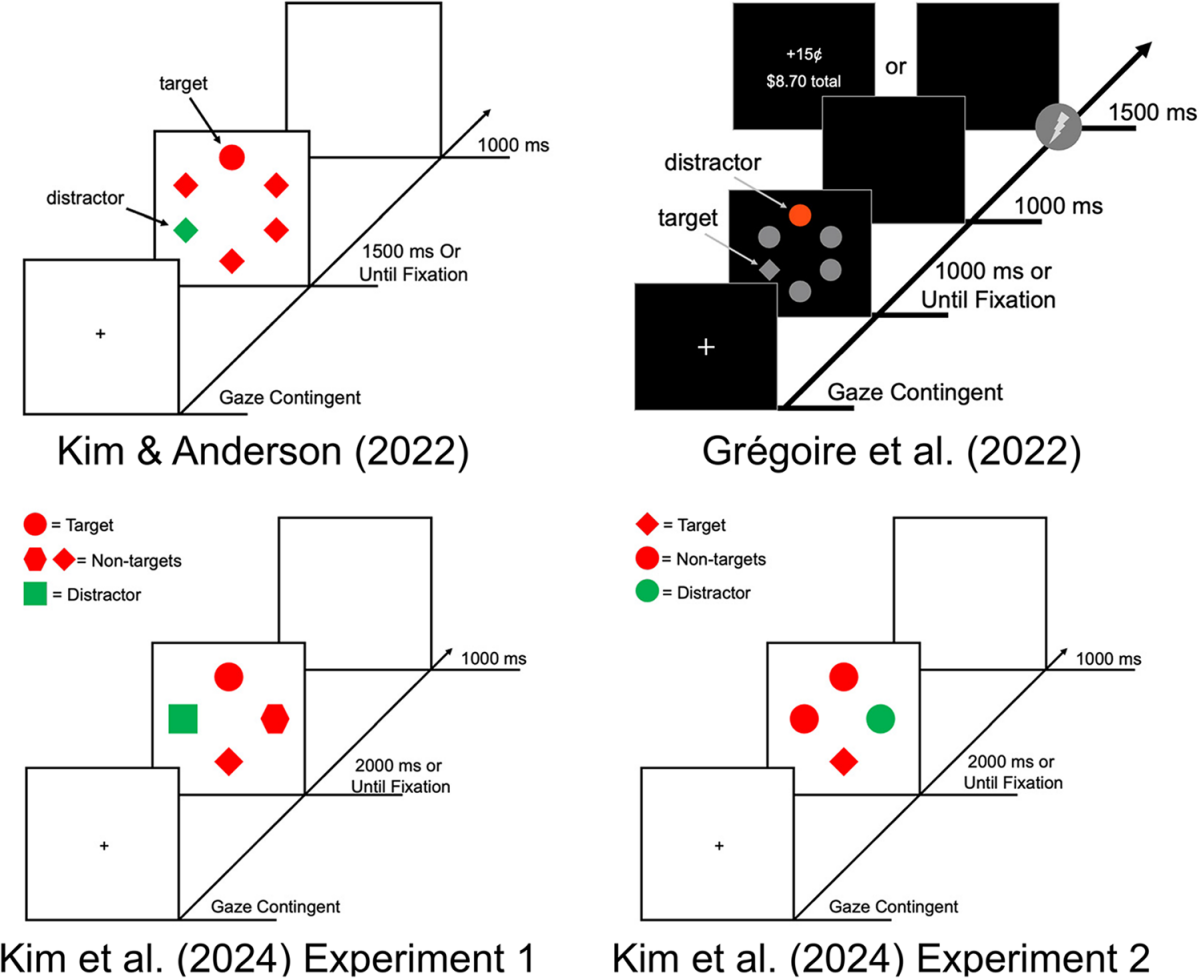
Sequence of trial events from the experiment used to generate each dataset with an example of a distractor-present trial. Although the stimuli used across datasets were similar, key differences in experimental design created a unique critical distractor that was used to probe different attentional processes

We evaluated three datasets that incorporated oculomotor measures in visual search tasks to investigate the reliability of oculomotor capture by the distractor and fixation times^1^ (oculomotor response times) between two critical distractor conditions (Grégoire et al., 2022; Kim et al., 2024; Kim & Anderson, 2022; see Table 1). In Kim and Anderson (2022), the critical distractor comparison was a distractor appearing at a high-probability location vs. a distractor appearing at a low-probability location (statistical learning of a high-probability distractor location; *n* = 36). In Grégoire et al. (2022), the critical distractor comparison was previously conditioned distractors (CS + ; associated with reward or electric shock) vs. neutral distractors (value- and threat-modulated attentional capture). In this latter study, we separated findings over the three experiments (focusing on the first two in which distractor suppression was observed; *n* = 38 for Experiment 1, *n* = 34 for Experiment 2, and *n* = 28 for Experiment 3). In Kim et al. (2024), the critical distractor comparison was attention capture by the distractor on distractor-present trials (first saccade to the distractor) vs. first fixation to a single non-target in distractor-absent trials (attention capture by a physically salient distractor when engaging in feature-search or singleton-search mode); reliability scores were separated by both experiments (feature-search vs. singleton-search) and calculated separately among young and older adult samples to probe potential age differences (*n* = 28 for all groups and experiments). For all experiments, each trial of the oculomotor visual search task ended when a fixation was made on the target (or no eye movement was registered to the target within the time limit). No manual response times were made in any experiment and an eye movement to the target was itself the required response. All experiments involved a search for a shape-defined target with some trials containing a salient, color singleton distractor (see Fig. 1).

**Table 1:** Critical experimental design components in analyzed datasets. The critical distractor condition in each experiment probes a unique attentional process explored in each manuscript. In Grégoire et al. (2022), participants learned associations between outcomes (reward, shock, neutral) and the distractor in a training phase. In addition, differences in the number of stimuli in the visual search array and the time limit across datasets may contribute to differences in reliability estimates that should be probed in future experiments

	Critical distractor condition	Training phase	Search array set size	Search array time limit
Kim and Anderson (2022)	Statistical learning of a high-probability distractor location	No	6	1500 ms
Grégoire et al. (2022)	Value- or threat-associated distractor	Yes	6	1000 ms
Kim et al. (2024) Exp. 1	Distractor suppression in feature search	No	4	2000 ms
Kim et al. (2024) Exp. 2	Attention capture by distractor in singleton search	No	4	2000 ms

#### Split-half reliability

Instead of utilizing an arbitrary odd vs. even split, we estimated internal consistency by utilizing a permuted random split procedure as in Garre-Frutos et al. (2024). In this procedure, all trials were randomly split into two halves with an equal number of observations in each half per condition per run to account for time-dependent effects (e.g., learning or extinction). Trials for each half were then concatenated over all runs. Then, a difference score between the two critical distractor conditions was computed for each concatenated half for each participant and correlated to get a Pearson’s *r* correlation coefficient. This procedure was repeated 1000 times, and the correlation coefficients were averaged to compute the mean split-half correlation. To examine the robustness of the acquired reliability measures, we converted each measure to a *z*-score and plotted histograms to test for the presence of outliers, of which there were only three across all experiments and measures (see Supplementary Fig. 1). All reliability measurements are reported with 95% confidence intervals. In addition, we also report Spearman–Brown-corrected (*r*_S-B_) reliability estimates using the following formula: *r*_*S-B* =_ 2*r* / (1 + *r*).

#### Non-parametric randomization tests

To determine whether estimates of reliability for oculomotor capture and fixation times were significantly different across conditions, we conducted non-parametric randomization tests. Based on the 1000 split-half correlation coefficients calculated for each measure (before averaging), we first computed the mean of the difference scores between the oculomotor capture and fixation time measures as the true sample mean. Then, from the combined 2000 coefficient values for both measures, we randomly assigned 1000 values to each measure to create two unique sample groups and computed the difference of these group mean *r* values (random sample), under the null hypothesis that there was no difference between split-half reliability obtained using each measure and, thus, random assignment of reliability to a dependent measure should tend to produce a similar difference score to the difference score observed between the two measures in the actual data. This randomization procedure was repeated 1000 times and the *p* value was manually calculated from the *z*-score using the observed sample mean.

## Results

### Kim and Anderson (2022)

**Fig. 2:**
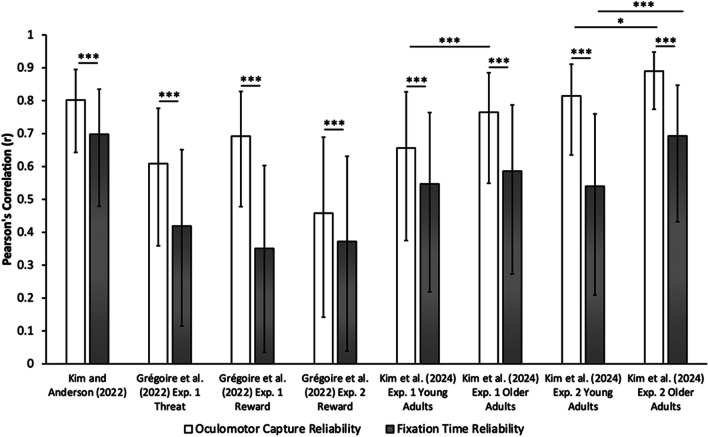
Split-half reliability of oculomotor capture is superior to reliability of fixation times. *Bar graphs* depict Pearson’s correlation values over attention capture by the distractor (oculomotor capture) and fixation times across multiple datasets. Regardless of critical distractor comparisons (high- vs. low-probability location; reward/threat-related vs. neutral; distractor-present vs. distractor-absent), type of visual search attentional template (feature-search vs. singleton-search), and age groups (young adults vs. older adults), the reliability of oculomotor capture was superior to the reliability of fixation times. Furthermore, the reliability of older adults was higher than that of young adults. *Error bars* reflect 95% confidence intervals of the Pearson correlation coefficient. *p < 0.05. ***p < 0.001

In Kim and Anderson (2022), visual search required fixating on a target shape singleton in the absence and presence of a salient color singleton distractor. Critically, the location of the color distractor in distractor-present trials was in a high-probability location 45% of the time and equally often in the other low-probability locations (five low-probability locations). When comparing the oculomotor measures, the split-half correlation for the learning-dependent reduction in oculomotor capture (probability of fixating the distractor on low-probability minus high-probability trials) was *r* = 0.802 [0.643, 0.895] (*r*_S-B_ = 0.890 [0.794, 0.943]) and for fixation time (latency to fixate the target on low-probability minus high-probability trials) was *r* = 0.698 [0.479, 0.835] (*r*_S-B_ = 0.822 [0.676, 0.906]). Using non-parametric randomization tests, we found that the reliability of oculomotor capture was significantly superior compared to fixation time, *p* < 0.001 (see Fig. 2)*.*

### Grégoire et al. (2022)

All three experiments in Grégoire et al. (2022) incorporated a paradigm that required participants to search for a unique shape singleton (circle among diamonds or diamond among circles), requiring participants to engage in singleton-search mode in the presence of color singleton distractors. Data from Experiments 1 and 2 were of particular interest given that reduced processing of valent (reward- and threat-related) distractors relative to neutral distractors was observed in these experiments. In contrast, the opposite was observed in Experiment 3, although reliabilities from all three experiments are reported for completeness. Data from both the training and test phases of each experiment were combined, given that mechanisms of attention capture by the distractor were identical in both phases, and the only difference in the test phase was the absence of feedback, which provided sufficient data to conduct a split-half analysis. Over all experiments, the critical distractor condition comparison was attention capture by the reward (Experiments 1–3) or threat-related distractor (Experiment 1 only) vs. the neutral distractor.

When comparing the difference in oculomotor measures between the threat-related vs. neutral distractor in Experiment 1, correlation values over the measure of oculomotor capture was *r* = 0.609 [0.359, 0.777] (*r*_S-B_ = 0.757 [0.577, 0.867]) and over fixation time was *r* = 0.419 [0.115, 0.651] (*r*_S-B_ = 0.591 [0.335, 0.766]). Like in Kim and Anderson (2022), we found that the reliability of the learning-dependent reduction in oculomotor capture was significantly superior compared to that observed using fixation time, *p* < 0.001*.* When comparing oculomotor measures between the reward-related vs. neutral distractor, the correlations between the critical distractor conditions over oculomotor capture were *r* = 0.692 [0.478, 0.828] (*r*_S-B_ = 0.818 [0.675, 0.902]) and *r* = 0.458 [0.142, 0.689] (*r*_S-B_ = 0.628 [0.368, 0.797]), and over fixation time were *r* = 0.351 [0.035, 0.603] (*r*_S-B_ = 0.520 [0.240, 0.720]) and *r* = 0.372 [0.039, 0.631] (*r*_S-B_ = 0.543 [0.251, 0.745]), across Experiments 1 and 2, respectively. Using non-parametric randomization tests, we again found that the reliability of the learning-dependent reduction in oculomotor capture was significantly superior compared to that observed using fixation time across both Experiments, *p*s < 0.001 (see Fig. 2)*.* Similar results were obtained in the context of oculomotor capture in the third experiment. However, overall reliability was somewhat reduced (*r* = 0.492 [0.146, 0.731] (*r*_S-B_ = 0.660 [0.381, 0.829]) for oculomotor capture and *r* = 0.272 [−0.112, 0.586] (*r*_S-B_ = 0.428 [0.065, 0.691]) for fixation time, *p* < 0.001).

### Kim et al. (2024)

In Experiment 1 of Kim et al. (2024), the task required searching for a specific target shape (circle or diamond, counterbalanced across participants), requiring participants to engage in feature-search mode, which generally promotes the suppression of salient distractors (Gaspelin & Luck, 2018; Gaspelin et al., 2015, 2017). We compared trials in which a salient color singleton distractor was present vs. absent (equally often) and separately for young adults (18–23 years old) and older adults (51–79 years old). Given that we measured attention capture by first fixations to the distractor on distractor-present trials, we summed the first fixations on non-targets in distractor-absent trials and divided the total by the number of non-targets in the visual search array to calculate the probability of fixating at any one non-target (proxy distractor on distractor-absent trials). When comparing oculomotor measures between these distractor conditions, correlations over oculomotor capture (probability of fixating a [proxy] distractor on distractor present vs. absent trials) were *r* = 0.656 [0.375, 0.827] (*r*_S-B_ = 0.792 [0.595, 0.899]) for young adults and *r* = 0.765 [0.549, 0.885] (*r*_S-B_ = 0.867 [0.730, 0.937]) for older adults while correlations over fixation times (latency to fixate the target on distractor present vs. absent trials) was *r* = 0.547 [0.219, 0.764] (*r*_S-B_ = 0.707 [0.454, 0.855]) for young adults and *r* = 0.586 [0.273, 0.787] (*r*_S-B_ = 0.739 [0.505, 0.872]) for older adults. Both young and older adults demonstrated superior reliability for oculomotor capture compared to fixation times, *p*s < 0.001 (see Fig. 2). In addition, older adults demonstrated superior oculomotor capture reliability compared to young adults, *p* < 0.001 (see Fig. 2). However, fixation time reliability was not significantly different between age groups, *p* = 0.229.

In Experiment 2, the task required searching for a unique shape singleton (circle among diamonds or diamond among circles), necessitating participants to engage in singleton-search mode. Under these conditions, attentional capture by the color singleton distractor is robust and difficult to suppress, requiring reactive distractor disengagement to complete the task (Bacon & Egeth, 1994; Geng, 2014; Theeuwes, 1992; Theeuwes et al., 1998). Again, we compared trials in which the distractor was present vs. absent (equally often) and separately for young adults (19–30 years old) and older adults (57–80 years old). When comparing oculomotor measures between these distractor conditions, correlations over oculomotor capture were *r* = 0.815 [0.635, 0.911] (*r*_S-B_ = 0.898 [0.789, 0.952]) for young adults and *r* = 0.890 [0.774, 0.948] (*r*_S-B_ = 0.942 [0.877, 0.973]) for older adults while correlations over fixation times were *r* = 0.540 [0.209, 0.760] (*r*_S-B_ = 0.701 [0.444, 0.851]) for young adults and *r* = 0.693 [0.432, 0.847] (*r*_S-B_ = 0.819 [0.642, 0.913]) for older adults. As in Experiment 1, both young and older adults demonstrated superior reliability for oculomotor capture compared to fixation times, *p*s < *0.0*01 (see Fig. 2). Furthermore, older adults demonstrated superior oculomotor capture reliability compared to young adults, *p* = 0.016, in addition to superior fixation time reliability, *p* < 0.001 (see Fig. 2).

## Discussion

Our findings demonstrate that, as a measure, oculomotor capture produces superior reliability compared to measures computed from fixation time across numerous critical distractor comparisons. Using the probability of fixating the distractor, reliable learning-dependent reductions in distractor processing can be observed (Grégoire et al., 2022; Kim & Anderson, 2022), in addition to a measure of attention capture that is reliable for both young and older adults regardless of whether capture is overall suppressed under conditions of feature search vs. singleton search. Even when accounting for the increased variance in difference score calculations (Miller & Ulrich, 2013; Paap & Sawi, 2016; Weichselbaum et al., 2018), we demonstrate that oculomotor measures of attention capture on average exhibit strong reliability (mean across acquired values, *r* = 0.711; *r*_S-B_ = 0.824) and are considerably more reliable than response time-based measures (Anderson & Kim, 2019; Freichel et al., 2023; Garre-Frutos et al., 2024; Ivanov et al., 2023).

Experimental psychologists have largely undervalued the utility of individual differences, and relationships between mechanisms of attentional control and other cognitive or self-report measures have been relatively unexplored. However, researchers investigating working memory capacity have examined individual differences to identify interactions between neural networks of memory and attention. Prior findings reveal that individuals with low working memory capacity exhibited stronger value-driven attentional capture (Anderson et al., 2011) and also took longer to disengage attention from a task-irrelevant distractor (Fukuda & Vogel, 2011). This relationship between working memory and attention is thought to be mediated by the locus coeruleus-noradrenaline system, particularly through modulation of the fronto-parietal attention networks (Unsworth & Robison, 2017). However, individual differences in working memory capacity were unable to predict performance in visual search tasks requiring feature or conjunction search (Kane et al., 2006). The lack of a relationship here is informed by the findings of Ivanov et al. (2023) in which attention capture and learning-dependent distractor suppression were investigated as potentially useful measures of individual differences using manual response times. Unfortunately, both within- and between-session reliability for both measures were poor despite robust group-level differences across conditions, suggesting that inconsistent findings relating individual differences in working memory capacity to attention may be due in part to the use of measures with poor reliability (all of the aforementioned studies and many similar studies used attention measures derived from manual response times). Interestingly, when value-driven attentional capture was measured from distractor fixations (Anderson & Yantis, 2012), the reported correlation with working memory capacity was numerically quite a bit stronger than when value-driven attentional capture was measured from manual response times (Anderson et al., 2011). Our findings suggest a potential path toward more consistent outcomes relating attention measures to other cognitive processes like working memory, and to the more fruitful exploration of individual differences in the learning-dependent control of attention more generally through fixation-based measures of attentional selection. More reliable measures of attentional control are of particular importance if the goal is to predict the progression of neurodegenerative diseases and other clinical outcomes, and our findings point to the value of eye tracking in the pursuit of such measures.

The set of experiments in Kim et al. (2024) additionally revealed that older adults exhibit greater reliability compared with young adults. Older adults generally have slower response times compared with young adults and this becomes problematic as overall slower response times have greater variability (Kim et al., 2024; Tse et al., 2010). Although Experiment 2 demonstrated that older adults make more first fixations to the distractor compared with young adults, superior reliability cannot be reduced to a product of this greater capture effect given that Experiment 1 showed similar oculomotor suppression by the distractor in both age groups but still greater reliability in older adults. The strong reliability of oculomotor measures in older adults can address a significant issue in the aging literature of low reliability due to increased error variance in measures like response time. Furthermore, the relatively higher reliability in Kim et al. (2024) suggests that the reliability of salience-driven capture may be higher compared with statistically learned distractor suppression (Grégoire et al., 2022; Kim & Anderson, 2022), which is in line with the results of Ivanov et al. (2023).

A natural question posed by the findings of the present study is why oculomotor capture produces a more reliable measure of distractor processing than fixation time in addition to what is typically observed in the literature with respect to manual response time. Although we can only speculate, this superior reliability may be found in the ballistic nature of the measure. Oculomotor capture essentially measures the probability that a task-irrelevant stimulus evokes greater attentional priority than the target at the time of saccade initiation, being directly linked to distractor-target competition in the visual system. Manual response time-based measures add a host of post-selection processes that are tied to target-response mappings and the execution of a manual response (often a keypress), all of which contribute variability that is removed when assessing oculomotor capture. Even in the context of fixation time, the time required to disengage attention from any non-target that is fixated and the efficiency with which the subsequent eye movement is targeted contribute additional variability that occurs after oculomotor capture is assessed, during which there is additional opportunity for task-unrelated processes (e.g., mind wandering) to randomly slow responses. If the goal is to measure distractor processing, the probability of initially fixating the distractor (oculomotor capture) may be the purest and most direct means of assessing it.

Our findings across multiple experiments suggest that the superior reliability of oculomotor capture relative to even response time-based measures derived from eye tracking may reflect a more general property of the measurements that would further generalize to other tasks and experimental situations. However, determining whether this is the case requires further investigation, in addition to the extent to which specific mechanisms of distractor processing (e.g., learning effects that promote capture vs. suppression, salience-driven vs. learning-dependent priority) are differently reliable. Similarly, it would also be important to investigate whether the observed high reliability of oculomotor capture as a measure extends to other mechanisms of distractor processing (e.g., contingent attention capture, emotion-modulated distraction).

The present study suggests a potential avenue forward for the field of psychological science to maximize reproducibility by utilizing oculomotor measures that exhibit high reliability. However, the biggest limitation in acquiring such measures is the accessibility of eye-tracking technology. All of the datasets analyzed utilized an EyeLink 1000 plus eye tracker (SR Research) that is far less accessible than what is required to conduct research using manual response time measures, both with respect to financial cost and training. The development of more reliable measures of visual information processing involving manual response time that can more closely approximate what we were able to achieve with oculomotor measures is therefore an important target for future research.

Another limitation is the sample size of the datasets we drew from in the present study and their ability to produce stable reliability estimates (Schönbrodt & Perugini, 2013). Based on Schönbrodt and Perugini’s (2013) calculations, our relatively high reliability estimates primarily provides robust stability despite the relatively low sample in the analyzed datasets. However, we recognize that some of our weaker correlations over the dependent measure fixation time may not be robust enough to provide stable estimates. While the level of confidence over the stability in our correlation estimates may not be integral to our specific research question in comparing reliability estimates of oculomotor capture and fixation time, a larger sample size may be required to provide higher confidence in the calculated reliability estimates. In addition, we did not conduct between-experiment comparisons of oculomotor reliability given the significantly different attentional processes probed by each experiment and the different experimental design features that may be contributing to reliability (see Table 1). Future experiments with standardized experimental designs may be beneficial to explore whether reliability between different distractor types (e.g., value-associated distractors vs. statistically learned probabilities) may be superior for conducting research on individual differences in distractor processing. Finally, our analysis of existing datasets limited our evaluations of reliability to internal consistency, and we were unable to explore stability over time (i.e., test–retest reliability; see Anderson & Kim, 2019). We urge the field to further explore the test–retest reliability of oculomotor measures to build the foundation for eye tracking to be a potential norm in individual differences research.

At least for the time being, until more reliable response time-based measures are developed, we recommend that researchers consider investing in oculomotor measures particularly when individual differences in distractor processing are of scientific interest. Oculomotor measures are naturally bound to experiments involving the processing of visual information, and it is also important to identify reliable measures of information processing in other sensory modalities in an effort to maximize statistical power and reproducibility.

## Supplementary information

Below is the link to the electronic supplementary material.

Supplementary file1 (DOCX 227 KB)

## Data Availability

The datasets analyzed during the current study and analysis scripts are available in the Open Science Framework repository, https://osf.io/fkj92/.

## Footnotes

1 When computing fixation times as a ratio (either between the two critical distractor conditions or a difference score between two different ratios involving distractor-absent trials), reliability estimates were comparable to when computed simply as a difference score between two conditions (maximum change was *.*078*)*. Statistical comparisons between fixation time vs. oculomotor capture did not change when computing as a ratio.

## Declarations

### Open practices statement

Materials are available at https://osf.io/fkj92/. None of the reported studies were preregistered.
