# Supplementary material for: Reliably measuring learning-dependent distractor suppression with eye tracking
Source: Behav Res Methods. 2024 Dec 18;57(1):18. doi: 10.3758/s13428-024-02552-8 (PMC11655588; doi:10.3758/s13428-024-02552-8)
Supplement: Supplementary file 1 — Supplementary file1 (DOCX 227 KB) [file 13428_2024_2552_MOESM1_ESM.docx]

**Supplementary Figure 1. Histograms to examine the robustness of the reliability measures.** For each participant, we calculated the mean capture scores for both oculomotor measures in the first split over 1000 iterations (given that the first and second split are conceptually identical). Then we converted each measure to a *z*-score and plotted histograms to examine each measure’s variability. Standard deviation refers to standard deviation from the mean. Across all experiments and measures, only three datapoints fell outside of 3 SD of the mean, in two cases for fixation time and in one case for oculomotor capture.
